# Hyperemesis Gravidarum Causing Wernicke's Encephalopathy and Korsakoff's Psychosis: A Case Report

**DOI:** 10.7759/cureus.44093

**Published:** 2023-08-25

**Authors:** Tajah M Alaithan, Lama K Alharbi, Saba M Aldusaymani, Tarig S Al Khuwaitir

**Affiliations:** 1 Medicine and Surgery, Al Maarefa University, Riyadh, SAU; 2 Internal Medicine, King Saud Medical City, Riyadh , SAU

**Keywords:** korsakoff's psychosis, wernicke's encephalopathy, thiamine deficiency, pregnancy, hyperemesis gravidarum, case report

## Abstract

Wernicke's encephalopathy (WE) is an acute neuropsychiatric emergency due to thiamine deficiency. This report includes a case of a young pregnant female in her second trimester (17th weeks) complaining of hyperemesis gravidarum, who, due to excessive vomiting, developed severe vitamin B1 deficiency, leading to WE and Korsakoff's psychosis. The typical triad of confusion, ocular signs, and ataxia is fundamental for the diagnosis of WE, yet not all cases present with the entire triad; however, our patient presented with all the symptoms. Replacement of thiamine, which is a crucial vitamin that plays an important role in the integrity of the nervous system, is the first step in the management of WE.

## Introduction

A severe thiamine insufficiency causes the condition known as Wernicke-Korsakoff syndrome (WKS) [1]. The prevalence of WKS is not as low as previously assumed. Alcoholics are more prone to have it. However, the prevalence ranges from 0.04% to 0.13% in non-alcoholics [1]. Seventy percent of pregnant women report experiencing nausea or hyperemesis between four and eight weeks and 14-16 weeks of their pregnancy [2]. During the first trimester, the patient may experience severe pregnancy-related hyperemesis, which necessitates hospitalization due to more than 5% weight loss, dehydration, electrolyte imbalance, and ketonuria. Extremely severe cases may also include immunological depression, WKS, liver and renal malfunction, and potentially the death of the patient and fetus [2]. To avoid a rare but fatal complication such as WKS, this case report emphasizes the significance of early identification, thiamin replacement, and careful management of hyperemesis gravidarum with a scientific approach [1].

## Case presentation

**Figure 1:**
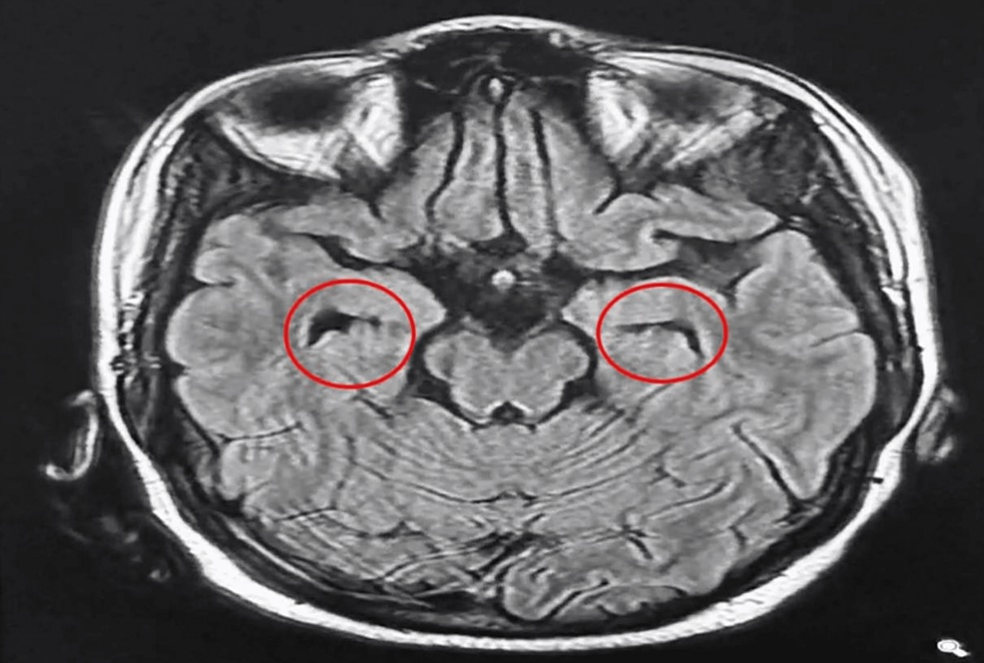
MRI brain without contrast showed mesial temporal lobes atrophic changes MRI: Magnetic Resonance Imaging

A 23-year-old Sudanese pregnant female (G1P0), in her second trimester (17 weeks), came to the emergency department, complaining of vomiting, dizziness, lower limb weakness, and visual and auditory hallucinations. She has a history of persistent vomiting for three months (hyperemesis gravidarum), a history of diarrhea, and no history of contact with a sick patient or similar complaints. Initially, she was admitted as a case of hypokalemia. In the emergency department, vital signs were conducted and revealed: RR: 20 bpm, pulse: 106 bpm, BP: 93/45 mmHg, T: 37 C, and pSo2: 95%. Her physical examination showed that a chest and abdominal examination were normal, apart from a palpable uterus. Neurological examinations showed an altered level of consciousness and drowsiness; the GCS was 13/15. Motor examination demonstrated upper and lower limb weakness; power of 4/5 and 1/5, respectively; and hypotonia in the lower limbs. Preserved reflexes and sensations were intact in all limbs. Cranial nerves displayed right third and sixth nerve palsies. Cerebellar signs in the lower limb were not performed due to patient incapability; no other significant findings were noted during the rest of the physical examination. Laboratory tests are previewed in (Table 1), and other laboratory results were unremarkable. MRI lumbar spine was obtained and was negative for disc bulge or herniation. MRI brain was obtained and showed mesial temporal lobes atrophic changes and no acute intracranial pathology (Figure 1). The ultrasound report showed appropriate fetal growth. She was diagnosed with a case of hypokalemia and temporary psychosis secondary to severe hyperemesis gravidarum and hypokalemia-induced periodic paralysis accompanied by Wernicke's encephalopathy due to thiamine deficiency. She was managed by NS IV, 1 L; omeprazole IV, 40 mg OD; vit B6, 40 ml oral TID; thiamine IV, 500 mg stat for three days and continued 250 mg TID for three days; 100 mg PO/OD; folic acid, 1 mg oral tablet for one week; metoclopramide IV, 10 mg TID; acetaminophen IV, 1,000 mg for 15 days; KCL oral, 600 mg TID; vit B complex PO, 100 mg TID; and enoxaparin SC 4000 international unit for one week. The hospital course lasted for 10 days, during which all appropriate management was performed, and all her sensorium and neurological symptoms were resolved especially after thiamine administration.

**Table 1:** Laboratory tests

Test	Result	Reference range
Hemoglobin (HGB)	10.8 (g/dL)	12-16 (g/dL)
Leukocyte count (WBC)	11.11(mm3)	5,000-10,000 (mm3)
Platelets count (PLT)	203 (x 109 /L)	152-472 (x 109 /L)
Prothrombin Time ( PT)	18.9 (seconds)	11.0-12.5 (seconds)
Activated partial thromboplastin time (aPTT)	30.3 (second)	30-40 (seconds)
International normalized ratio (INR)	1.41(seconds)	0.8-1.1 (seconds)
Potassium (K)	2.9 (mEq/L)	3.5-5.0 (mEq/L)
Thyroxine (FT4 )	11.68 (pmol/L)	9-23 (pmol/L)
Triiodothyronine (FT3 )	3.82 (nmol/L)	1.2-3.4 (nmol/L)
Thyroid-stimulating hormone (TSH)	0.015 (μU/mL)	2-10 (μU/mL)
Aspartate aminotransferase (AST)	93.5 (units/L)	0-35 (units/L)
Alanine aminotransferase (ALT)	36.6 (units/L)	4-36 (units/L)
Alkaline phosphates	150 (U/L)	44-147 (U/L)
Direct bilirubin	7.3 (μmol/L)	1.7-5.1 (μmol/L)
Serum sodium (Na)	143 (mmol/L)	135-147 (mmol/L)
Calcium (Ca)	2.80 (mg/dL)	4.5-5.6 (mg/dL)
Phosphorus (P)	0.54 (mmol/L)	0.97-1.45 (mmol/L)
Creatinine	42 (μmol/L)	44-97 (μmol/L)
Urea	3.8 (mg/dL)	2.7-7.3 (mg/dL)
Blood urea nitrogen (BUN)	18.7 (mg/dL)	10-20 (mg/dL)
Antinuclear antibody (ANA)	Negative	(< 1.0 IU) is negative
Cobalamin (vit B12)	306.6 (pg/ml)	160-950 (pg/ml)

## Discussion

A presentation that goes back to 2000 BC, early literature made it apparent that mild illness and nausea were unlikely to be dangerous and to be spontaneously resolved by the end of the fourth month of gestation. In 1852, Dubois was the first to describe the classical clinical signs of pernicious vomiting [3].

Hyperemesis gravidarum is a complication of early pregnancy distinguished by persistent uncontrollable nausea and vomiting strong enough to produce weight loss, dehydration, and electrolyte disturbance that can be life-threatening and, in rare cases, to neuropsychiatric conditions known as WKS. It is a fatal complication that results from severe thiamine deficiency [1]. Thiamine is an essential cofactor in the metabolism of energy, and delaying treatment causes irreversible gliosis and neuronal necrosis, particularly in the medial dorsal nuclei. This shows up as a partial recovery of memory impairments. However, the quick reversibility of ocular symptoms points to a biochemical, rather than a morphological, anomaly in the sixth and third nerve nuclei and vestibular nuclei [4].

Our patient presented with the typical triad of confusion, ocular signs, and ataxia, which follows the new criteria for diagnosis of WKS, which requires two of the following four signs: (i) dietary deficiencies, (ii) oculomotor abnormalities, (iii) cerebellar dysfunction, and (iv) either an altered mental state or mild memory impairment [1]. However, most cases may not display the full spectrum of clinical abnormalities [5]. The diagnosis could easily have been overlooked. Therefore, it is necessary to investigate each patient with hyperemesis gravidarum using liver and thyroid function tests. Our case highlights the significance of early detection of this relatively uncommon illness in the unique context of HG. Vitamin B deficiency is common in alcoholic liver disease, in particular deficiency of thiamine, folate, pyridoxine, and riboflavin. Thiamine deficiency also causes depletion of ATP in the liver, which may result in elevated liver enzymes, which is why it is very crucial to recognize the signs of WKS and treat them as early as possible due to its unfavorable consequences, such as liver and thyroid dysfunction. Therefore, it is fundamental to investigate each patient with hyperemesis gravidarum using liver and thyroid function tests [1].

## Conclusions

In conclusion, WE is a relatively uncommon condition, and it might be misdiagnosed especially if it happens due to hyperemesis gravidarum during pregnancy period. Therefore, it is extremely important for the physician to have a high index of suspicion of any of the signs and symptoms of WE, especially if it is accompanied by hyperemesis gravidarum. Early recognition and good awareness will facilitate better outcomes. Electrolytes, along with thiamine, warranted the prevention of permanent neurological deficits.

## References

[1] Mishra VV, Verneker RA (2019). Hyperemesis gravidarum causing Wernicke-Korsakoff syndrome: a rare complication. J Obstet Gynaecol Res.

[2] Levine MG, Esser D (1988). Total parenteral nutrition for the treatment of severe hyperemesis gravidarum: maternal nutritional effect and fetal outcome. Obstetrics Gynecology.

[3] Davis CJ, Lake-Bakaar GV, Grahame-Smith DG (1986). Nausea and Vomiting: Mechanisms and Treatment.

[4] Kleihues P (1992). Greenfield's neuropathology Fifth Edition, Edited by J.H. Adams and L.W. Duchen. Brain Pathology.

[5] Harper CG, Giles M, Finlay-Jones R (1986). Clinical signs in the Wernicke-Korsakoff complex: a retrospective analysis of 131 cases diagnosed at necropsy. J Neurol Neurosurg Psychiatry.

